# Niche breadth and divergence in sympatric cryptic coral species (*Pocillopora* spp.) across habitats within reefs and among algal symbionts

**DOI:** 10.1111/eva.13762

**Published:** 2024-08-02

**Authors:** Scott C. Burgess, Alyssa M. Turner, Erika C. Johnston

**Affiliations:** ^1^ Department of Biological Science Florida State University Tallahassee Florida USA; ^2^ Present address: Hawai‘i Institute of Marine Biology Kāne‘ohe Hawaii USA

**Keywords:** cryptic species, molecular ecology, Moorea, niche partitioning, symbiosis, sympatry

## Abstract

While the presence of morphologically cryptic species is increasingly recognized, we still lack a useful understanding of what causes and maintains co‐occurring cryptic species and its consequences for the ecology, evolution, and conservation of communities. We sampled 724 *Pocillopora* corals from five habitat zones (the fringing reef, back reef, and fore reef at 5, 10, and 20 m) at four sites around the island of Moorea, French Polynesia. Using validated genetic markers, we identified six sympatric species of *Pocillopora*, most of which cannot be reliably identified based on morphology: *P. meandrina* (42.9%), *P. tuahiniensis* (25.1%), *P. verrucosa* (12.2%), *P. acuta* (10.4%), *P. grandis* (7.73%), and *P.* cf. *effusa* (2.76%). For 423 colonies (58% of the genetically identified hosts), we also used *psbA* ^ncr^ or ITS2 markers to identify symbiont species (Symbiodiniaceae). The relative abundance of *Pocillopora* species differed across habitats within the reef. Sister taxa *P. verrucosa* and *P. tuahiniensis* had similar niche breadths and hosted the same specialist symbiont species (mostly *Cladocopium pacificum*) but the former was more common in the back reef and the latter more common deeper on the fore reef. In contrast, sister taxa *P. meandrina* and *P. grandis* had the highest niche breadths and overlaps and tended to host the same specialist symbiont species (mostly *C. latusorum*). *Pocillopora acuta* had the narrowest niche breadth and hosted the generalist, and more thermally tolerant, *Durusdinium gynnii*. Overall, there was a positive correlation between reef habitat niche breadth and symbiont niche breadth—*Pocillopora* species with a broader habitat niche also had a broader symbiont niche. Our results show how fine‐scale variation within reefs plays an important role in the generation and coexistence of cryptic species. The results also have important implications for how niche differences affect community resilience, and for the success of coral restoration practices, in ways not previously appreciated.

## 1 INTRODUCTION

The ability of biological communities to adjust to changing environmental conditions, and the success of conservation and management interventions, depends on there being a portfolio of species and genomes that promote resilience and adaptive potential (Baums et al., 2022; Colton et al., 2022; Matz et al., 2020; McManus et al., 2021; Webster et al., 2017). A critical and non‐trivial aspect of this endeavor is the organization of genetic diversity into species (Bickford et al., 2007; Pante et al., 2015; Ramírez‐Portilla et al., 2022; Sheets et al., 2018). Genomic techniques over the past few decades have drastically reshaped our understanding of species boundaries and their permeability to gene flow in natural populations (Bridge et al., 2023; Cowman et al., 2020; Fišer et al., 2018; Stanton et al., 2019). Especially in the case of corals, cryptic species are being rapidly discovered using genomic techniques that have identified evolutionarily distinct lineages, as well as hybrid or introgressed lineages that are not always congruent with current species descriptions relying on morphological differences (e.g., Bongaerts et al., 2021; Bridge et al., 2023; Oury et al., 2023; Pinzón et al., 2013; Schmidt‐Roach et al., 2014; Voolstra et al., 2023). Incorrectly grouping morphologically similar individuals from genetically divergent lineages will provide misleading estimates of biodiversity, population connectivity, and adaptive potential, which in turn makes it challenging to make scientifically informed decisions about coral conservation and restoration. While the presence of morphologically cryptic species is increasingly recognized, we still lack a useful understanding of what causes and maintains co‐occurring cryptic coral species within reefs. Specifically, we need to know the diversity of resources used and environments tolerated (niche breadth) and the extent to which these differ among species (niche overlap), as well as differences in the identity of symbionts that could underlie holobiont adaptation.

One of the main hypotheses for the generation and coexistence of cryptic species is that different habitats create strong spatially varying selection that results in environmental specialization and niche shifts among species (e.g., Bongaerts et al., 2013, 2021; Carscadden et al., 2020; De Meester et al., 2015; Derycke et al., 2016; Dionne et al., 2017; Johnston, Wyatt, et al., 2022; Kenkel et al., 2013; Matias et al., 2023; Prada & Hellberg, 2014; Rippe et al., 2021; Sturm et al., 2022; van Oppen et al., 2018; Wellborn & Cothran, 2007). A ubiquitous feature of coral reefs with potential to create such strong spatially varying selection is the underlying reef structure. The physical structure of a coral reef often comprises contiguous and distinct habitat zones, such as the fringing reef, shallow back reef lagoon, reef crest, and different depths on fore reef slope (Goreau, 1959; Huston, 1985). These habitat zones are well known to differ in multiple environmental factors such as light, turbidity, wave energy, current speed, temperature, and nutrients, and to comprise distinct coral reef communities as a result (Burgess et al., 2010; Connolly et al., 2005; Guadayol et al., 2014; Johnston, Wyatt, et al., 2022; Karlson & Cornell, 1998; Pérez‐Rosales et al., 2022; Reid et al., 2019; Wyatt et al., 2020, 2023). Habitat zones are also differentially affected by disturbances, such as heatwaves, cyclones, coralivorous Crown‐of‐Thorns seastar outbreaks, land‐based pollution, and over‐fishing (Baird et al., 2018; Crosbie et al., 2019; Donovan et al., 2020; Penin et al., 2007; Sully & van Woesik, 2020). The extent to which some habitats can act as a temporary micro‐refuge within reefs, and the vulnerability of certain species to disturbances, depends on how cryptic species are distributed across such habitats within reefs.

Niche breadth and overlap of cryptic species could also depend on the identity of the photosynthetic microalgae from the family Symbiodiniaceae with which they form an obligate symbiosis (Buitrago‐López et al., 2023; Glynn et al., 2023; Gómez‐Corrales & Prada, 2020; Hoadley et al., 2021; Kemp et al., 2023; Marzonie et al., 2024; Palacio‐Castro et al., 2023; Parkinson & Baums, 2014; Starko et al., 2023; Thornhill et al., 2014; Turnham et al., 2021). Much of the coral's nutrition relies on the photosynthetic algae to convert inorganic carbon and nitrogen to nutrients through photosynthesis and absorption of nitrogen wastes. The environmental sensitivity of this feedback may differ among algal species and genotypes, or among specific combinations of coral host and algal species or genotypes to influence holobiont fitness (Cornwell et al., 2021; Hoadley et al., 2021; Kemp et al., 2023; Starko et al., 2023; Wall et al., 2020). Because symbiotic Symbiodiniaceae live inside coral cells, they are identified using genetic markers (LaJeunesse et al., 2018). Historically there has been a lack of consensus regarding the organization of genetic data into species, which has slowed progress on understanding how the environment shapes the distribution of symbiont and host genetic diversity, but recent advances in Symbiodiniaceae genomics have led to a growing consensus on how to interpret the genetic diversity in this group (Davies et al., 2023).

A particularly valuable system to understand the environmental sensitivity of morphologically similar yet genetically divergent species is corals in the genus *Pocillopora*. Pocilloporid corals are distributed across contiguous habitat zones within reefs in the Indo‐Pacific, and often dominate reefs in terms of percent cover (Edmunds et al., 2016; Pérez‐Rosales et al., 2021). Our previous work at Moorea, French Polynesia, uncovered five co‐occurring broadcast spawning Pocilloporid species in the fore reef habitat alone that can only be identified reliably using verified genetic markers (Johnston, Cunning, et al., 2022). These cryptic species differed in their relative abundance on the fore reef slope across depths of only 5, 10, and 20 m (Johnston, Wyatt, et al., 2022). Differences in relative abundance across depths on the relatively steep fore reef slope (all accessible in a single dive) were greater than differences among sites separated by several kilometers.

Therefore, we hypothesized that the well‐known environmental differences among structural habitat zones typical within coral reefs are strong enough to generate niche differences among cryptic *Pocillopora* species, and this would be reflected by different relative abundances of host species among habitats and the identity and diversity of the photosynthetic algal symbionts they host. We sampled *Pocillopora* corals across five habitats (the fringing reef, back reef, 5 m on the fore reef, 10 m on the fore reef, and 20 m on the fore reef slope) extending seaward from the shore at each of four sites separated by several kilometers around the island of Moorea, French Polynesia. Specifically, we (1) quantified the relative abundance of genetically identified *Pocillopora* species across habitats, (2) tested for associations between coral host (*Pocillopora*) and symbiont (Symbiodiniaceae) identity, (3) tested how habitat type affected genetic variation in symbionts, and (4) quantified niche breadth and divergence among *Pocillopora* species and the extent to which habitat niche breadth correlated with symbiont niche breadth among *Pocillopora* species.

## 2 METHODS

### 2.1 Sites and sampling design

**FIGURE 1:**
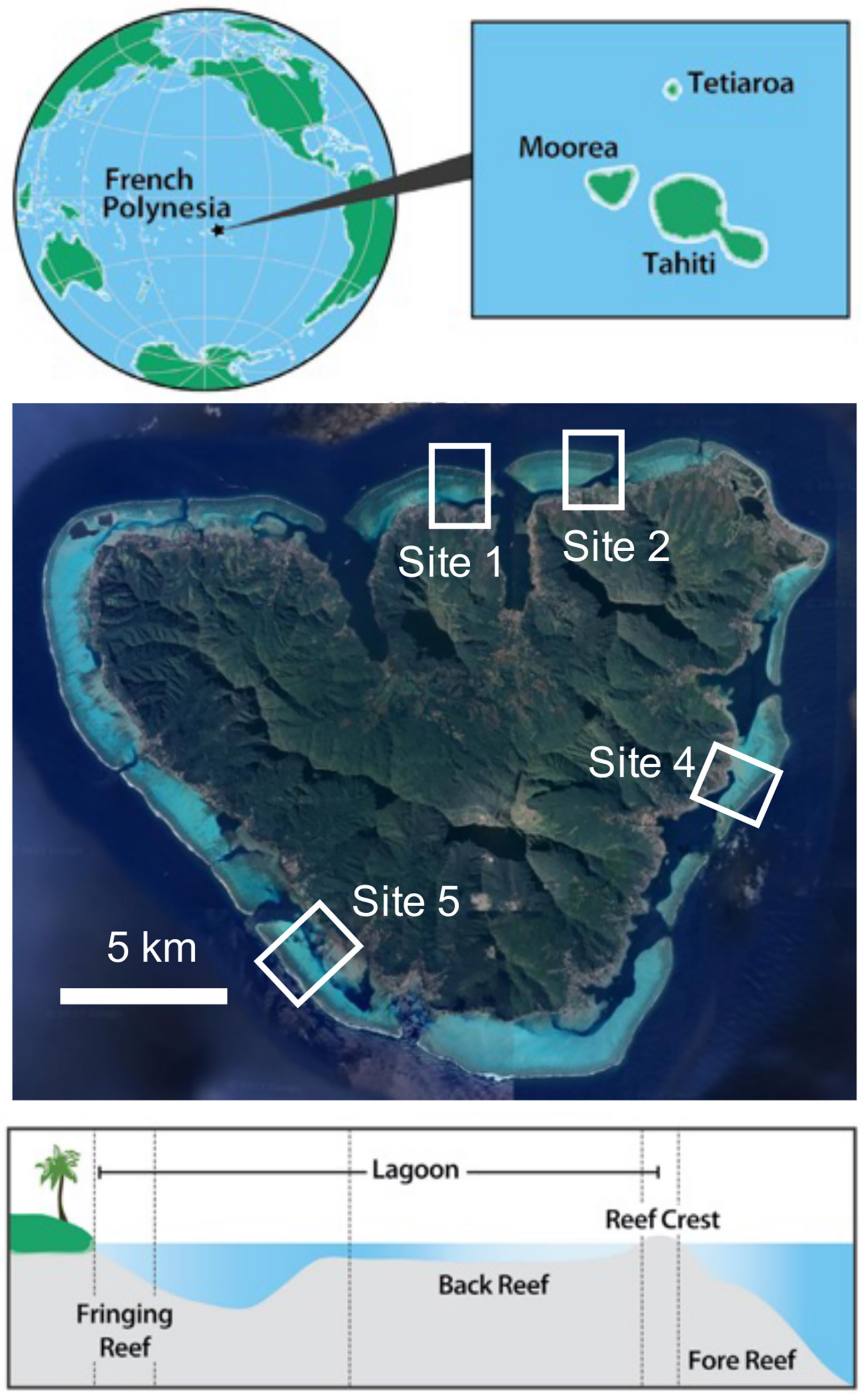
Map of Moorea (French Polynesia) showing the location of sites 1, 2, 4, and 5. Within each site, five habitats were sampled: Fringing reef (1 m), back reef (2 m), fore reef 5 m, fore reef 10 m, and fore reef 20 m. Figure adapted from Leichter et al. (2013).

We sampled *Pocillopora* colonies from five different habitats at each of four sites (sites 1, 2, 4, and 5) of Moorea (Figure 1). Site locations and names correspond to those used by the Moorea Coral Reef Long‐Term Ecological Research (MCR‐LTER) program (Holbrook et al., 2018). At each site, the five habitats were: (1) fringing reef, (2) back reef lagoon, (3) fore reef at 5 m depth, (4) fore reef at 10 m depth, and (5) fore reef at 20 m depth. The fringing reef sites were ~1 m deep and ~20–30 m from shore at sites 1 and 2, 60 m from the shore at site 4, and 140 m from the shore at site 5. The back reef lagoon sites were ~1–2 m deep and typically ~600–900 m from the shore. The fore reef sites have slope angles ranging from 33% to 55% among sites (Leichter et al., 2012) and were typically 1000–1200 m from shore.

At each habitat at each site (except the fore reef 10 m), 50 cm × 50 cm quadrats (as many as was logistically possible) were randomly placed on the reef along the target depth contour to estimate relative abundance. The samples from the fore reef 10 m habitat came from colonies whose tissue was sampled in 2019 (24 70 cm × 70 cm quadrats per site) from Burgess et al. (2021) and were confirmed to still be alive in censuses conducted in December 2021 (hence smaller sample sizes). Except for the fore reef 10 m, samples from all habitats at sites 1 and 2, and from the fore reef at sites 4 and 5 were collected in December 2021. Samples from the fringing reef and back reef lagoon at sites 4 and 5 were collected in December 2022. There was no obvious disturbance (e.g., COTS, cyclone) or environmental event (e.g., marine heatwave) between December 2021 and December 2022. Only samples collected in December 2021 were used to assess symbiont identities, to avoid the potential influence of different sampling periods on symbiont assemblages. Tissue (~1 cm) from every *Pocillopora* colony within the quadrat was sampled (Table 1), which meant we did not target specific morphologies that could bias results. Tissue was collected from a total of 724 *Pocillopora* colonies. Tissue was collected using small bone clippers and stored in DMSO buffer or DNA/RNA Shield (Zymo Research).

**TABLE 1:** The number of samples analyzed using mtORF (for *Pocillopora* species identification), ITS2 (for Symbiodiniaceae identification), and *psbA* ^*n*cr^ (for *Cladocopium* species identification) in each habitat and site.

Habitat	Site 1	Site 2	Site 4	Site 5	Total
Fringing reef (1 m)	20/20/5	35/35/12	50/0/0	49/0/0	154/55/17
Back reef (2 m)	61/51/46	60/51/41	50/0/0	50/0/0	221/102/87
Fore reef (5 m)	35/31/21	36/31/25	50/34/29	33/31/30	154/127/105
Fore reef (10 m)	20/0/0	17/0/0	14/0/0	11/0/0	62/0/0
Fore reef (20 m)	35/18/35	35/27/30	37/24/30	26/24/22	133/93/117

*Note*: Site numbers correspond to those used for the MCR LTER. Numbers are presented in the following format: mtORF/ITS2/*psbA* ^ncr^.

### 2.2 DNA extraction and quantification

Genomic DNA was extracted from tissue using the OMEGA (Bio‐Tek) E.Z.N.A. Tissue DNA Kit or Chelex 100 Resin (Bio‐Rad). OMEGA DNA extractions were used for all samples from December 2021 and processed for the genetic identification of Symbiodiniaceae. For the OMEGA extractions, three elutions (50, 100, and 100 μL) were collected in HPLC grade H2O. After inspection on a 1% agarose gel, all three elutions were combined. Extractions were quantified using the Qubit dsDNA HS Assay kit with the Qubit Fluorometer (ThermoFisher Scientific). For the Chelex extractions, 10 μL of DNA Shield from which the sample was preserved was placed into 100 μL of 10% Chelex solution and incubated at 55°C for 60 min followed by 95°C for 20 min.

### 2.3 Genetic identification of *Pocillopora* species

Extracted DNA was used for PCR amplification using the mitochondrial Open Reading Frame (mtORF) marker, using the FATP6.1 and RORF primers detailed in Flot et al. (2008). The mtORF region corresponds to a protein‐coding gene (*tmp362*) that is hypothesized to have functional effects on metabolic functions and thermal adaptation (Banguera‐Hinestroza et al., 2019). PCR mixes contained 5 μL of BioMix Red (Bioline Ltd., London, UK), 0.13 μL of each forward and reverse primer, 0.1 μL of BSA, 1 μL of template DNA (5–20 ng/μL), and 3.64 μL of deionized water to 10 μL final volume. Each PCR followed the cycling protocol of Flot et al. (2008), with a denaturation step of 60 s at 94°C, followed by 40 cycles (30‐s denaturation at 94°C, 30‐s annealing at 53°C, and 75‐s elongation at 72°C), and a final 5‐min incubation at 72°C for 5 min. Five microliters of PCR product was cleaned by incubating with 2 μL of Exo‐CIP Rapid (New England BioLabs, Ipswich. Massachusetts), and placed in a thermocycler for 4 min at 37°C and 1 min at 80°C. Cleaned PCR products were sequenced in the forward direction with the FATP6.1 primer at Florida State University on an Applied Biosystems 3730 Genetic Analyzer with Capillary Electrophoresis. In Geneious Prime v2024.0.5, forward mtORF sequences (~800 bp) were aligned with reference sequences (GenBank Accessions FR729326.1, HQ378759.1, HQ378760.2, HQ378761.1, JX994075.1, JX994085.1, JX994083.1, JX994079.1, JX994072.1, JX994073.1, JX994074.1, OP418359.1, KF381328.1, and EF633600.2 [outgroup]) using Clustal Omega 1.2.3. Samples were identified to haplotype based on similarity to reference sequences of mtORF haplotypes, using the naming conventions in Pinzón et al. (2013), Forsman et al. (2013), and Johnston, Cunning, et al. (2022).

*Pocillopora meandrina* and *P. grandis* both share mtORF haplotype 1, but are distinct species (Johnston et al., 2017; Johnston, Cunning, et al., 2022), so to distinguish these two species we used a restriction fragment length polymorphism (RFLP) gel‐based assay following Johnston et al. (2018). DNA from samples of mtORF haplotype 1 was used for PCR amplification of the histone 3 region using the PocHistone primers PocHistoneF and PocHistoneR detailed in Johnston et al. (2018). PCR mixes were prepared as described above using the following thermocycling protocol: an initial denaturation step of 60 s at 94°C, followed by 40 cycles of 30 s at 94°C, 30 s at 53°C, and 60 s at 72°C, and a final 5‐min extension step at 72°C. Five microliters of PCR product was then digested with 0.5 μL of *XhoI* restriction enzyme (New England BioLabs, Ipswich, MA, USA), 0.8 μL of 10× CutSmart buffer, and 2.7 μL of deionized water, for a final volume of 9 μL for 1 h at 37°C, followed by heat inactivation for 20 min at 65°C (Johnston et al., 2018). Three microliters of the digested products were run on a 1% or 2% TAE‐agarose gel at 70 V. Samples that are *P. meandrina* present as a single band on the gel around ~700 bp, while samples that are *P. grandis* present as two bands around ~400 and 300 bp. Sometimes *P. grandis* presents as three bands (at ~700, ~400, and ~300 bp), and our sequencing of the histone 3 region consistently confirms that these samples are *P. grandis*, and that three bands likely arises from incomplete cutting of the restriction enzyme.

Importantly, we have previously validated the use of the mtORF and histone 3 markers as suitable species‐level markers to delineate *Pocillopora* species at Moorea (Johnston, Cunning, et al., 2022). In that study, we used multiple genomic datasets (complete mitochondrial genomes, nuclear genomic loci, and thousands of linked and unlinked genome‐wide single nucleotide polymorphisms) and phylogenetic approaches (Bayesian phylogenetic trees, SNAPP species trees, and discriminant analysis of principal components), in combination with algal symbiont identity and distribution patterns, to identify distinct evolutionary lineages of *Pocillopora* that are considered to be taxa on the basis of genetically distinct groups occurring in sympatry (De Queiroz, 2007). At Moorea, mtORF haplotype 1 *P. meandrina* and haplotype 8, despite being distinct mitochondrial lineages (differing by seven base pairs), are not differentiated when using (nuclear) genome‐wide single nucleotide polymorphisms (SNPs) (Johnston, Cunning, et al., 2022). Therefore, colonies identified as mtORF haplotype 8 were included with colonies identified as *P. meandrina*, which was also justified based on their similar within‐reef distribution patterns and responses to heatwaves (Burgess et al., 2021; Johnston, Cunning, et al., 2022). Our organization of mtORF haplotypes into taxa based on genome‐wide data is consistent with subsequent studies (Oury et al., 2023; Voolstra et al., 2023) that have also confirmed the suitability of the mtORF marker (plus histone marker in the case of mtORF haplotype 1) using complementary species delimitation approaches on genome‐wide SNPs (Table 2).

**TABLE 2:** Summary of the mtORF haplotypes that were used to identify *Pocillopora* species, and their associated names, following Johnston, Cunning, et al. (2022).

Species name	mtORF haplotype	Oury et al. (2023) Genomic species hypotheses (GSH)	Voolstra et al. (2023) SVDquartet lineage
*P. tuahiniensis* ^a^	10	14	SVD5
*P. meandrina*	1a (+ PocHistone)	09a,b	SVD2
	8a	09a,b	
*P. grandis*	1a (+ PocHistone)	09c	SVD4
*P. verrucosa*	3a		SVD3
	3b, 3f, 3 h	13c	
	3e	13a	
*P*. cf. *effusa*	2	01	SVD1
	11		
*P. acuta*	5a	05	‐

*Note*: The association of mtORF haplotypes and species names with species hypotheses identified in other studies using genome‐wide data is also provided for comparison and consistency across studies.

^a^ Taxonomic description in Johnston and Burgess (2023).

### 2.4 Genetic identification of Symbiodiniaceae

Extracted DNA from 377 samples was used for PCR amplification of the ribosomal internal transcribed spacer 2 (ITS2) region using the SYM_VAR primer pairs (Hume et al., 2018). Initial PCR was performed using Phusion High‐Fidelity MasterMix (ThermoFisher Scientific) with an initial denaturation step at 98°C for 2 min, followed by 35 cycles of 98°C for 10 s, 56°C for 30 s, and 72°C for 30 s, and a final extension step at 72°C for 5 min. Amplified DNA was cleaned using AMPure XP beads and used as template for an index PCR in which unique combinations of Nextera XT index primers were used for each sample. Eight cycles of PCR were performed as described above with an annealing temperature of 55°C. Libraries were then cleaned, normalized, and pooled for sequencing on an Illumina MiSeq platform with 2× 300 paired‐end reads at Florida State University. We included four technical replicates (i.e., four samples where the PCR was performed twice), one on each 96‐well plate.

Demultiplexed forward and reverse fastq files were passed remotely to SymPortal (Hume et al., 2018), which removed non‐Symbiodiniaceae sequences and then grouped Symbiodiniaceae sequences by genera. Being a ribosomal gene, there are usually multiple ITS2 copies within a single Symbiodiniaceae cell. ITS2 sequence variation can, therefore, come from intragenomic variation among gene copies, in addition to intergenomic variation among Symbiodiniaceae genotypes. To parse ITS2 sequence variation into intra‐and intergenomic variation, SymPortal identifies repeatedly co‐occurring sequences with similar relative abundances as “defining intragenomic variants” (DIVs), which are then collapsed into ITS2‐type profiles that may represent distinct taxonomic units above, at, or below the species level (Hume et al., 2018). The full (post‐quality filtering) diversity of ITS2 sequence variation was used for downstream statistical analysis, while type profiles were used to aid visualization only.

Since ITS2 is not a species‐level marker, we also used the non‐coding plastid minicircle (*psbA* ^ncr^) to distinguish between *Cladocopium* species (Moore et al., 2003) for 326 *Pocillopora* colonies (Table 1). The *psbA* ^ncr^ region was amplified using the 7.4‐Forw and 7.8‐Rev primers and protocol of Moore et al. (2003), and the region was sequenced in both the forward and reverse directions. Sequences were aligned and manually checked in Geneious Prime. To place the clades in our current study, we included five sequences from Johnston, Cunning, et al. (2022), one from each of the *Cladocopium* clades found in that study, and as outgroups included two sequences of *C. goreaui* from Turnham et al. (2021) and three sequences of *Cladocopium* spp. collected from *Pocillopora* cf. *effusa* colonies from Clipperton Atoll (Pinzón & LaJeunesse, 2011). A Bayesian phylogeny of this larger data set was generated using beast version 2.6.2 (Bouckaert et al., 2019). We used the GTR model of evolution, a random local clock, and the birth‐death model as the tree prior. The MCMC was run for 100,000,000 generations with sampling every 5000 steps and the first 20% was removed as burnin. Generally, clades within *C. latusorum* and *C. pacificum* were identified by high posterior support (>95%) at deep monophyletic nodes, though divergence between these clades was greater in *C. latusorum* than in *C. pacificum*.

In summary, we identified symbionts in 423 colonies, which had ITS2 sequences, *psbA* ^ncr^ sequences, or both (280 samples had both ITS2 and *psbA* ^ncr^ sequences). Out of the 377 samples with ITS2 sequences, 97 (26%) did not have *psbA* ^ncr^ sequences. Out of the 326 samples with *psbA* ^ncr^ sequences, 46 (14%) did not have ITS2 sequences.

### 2.5 Statistical analyses

All analyses were done in R v4.4.0. To quantify how the relative abundance of a given species differed across environments, we first assigned a colony a value of 1 if it was the focal species, or 0 if it was not the focal species. Then, we fit binomial generalized linear models (glm) using glmmTMB v1.1.9 (Brooks et al., 2017) with a logit link function and habitat as a fixed effect to test if the proportion of colonies belonging to a given species in each habitat (i.e., relative abundance of that species) varied across habitats. Model residual diagnostics were assessed using DHARMa v0.4.6 (Hartig, 2022). To test for the effect of habitat, we used log‐likelihood ratio tests where the deviance was assessed against a *χ* ^2^ distribution. Because not every species was found in every combination of habitat and site, we could not statistically assess the interactive or additive effects of site on the patterns of relative abundance across habitats within each species. As a result, sites were pooled for the binomial glm, but the raw proportions presented in Figure S1 show similar relative abundance patterns across sites.

To analyze variation in the composition of *Pocillopora* species among habitats, we used distance‐based redundancy analysis (dbRDA) implemented in the vegan v2.6–6.1 package in R (Oksanen et al., 2022). The principal coordinates were estimated using Bray–Curtis dissimilarity matrices (based on the proportion of individuals belonging to each species in each site and habitat). Habitat was the constraining variable. The effects of habitat were determined using an ANOVA‐like permutation test with 99,999 permutations implemented in anova.cca. Ordination biplots were used to visualize results and aid in interpretation.

To visualize differences in Symbiodiniaceae ITS2 sequence diversity between *Pocillopora* species, we used principal coordinate analyses (PCoA) implemented using cmdscale function in the R package vegan with a Bray–Curtis dissimilarity measure. Differences among species were tested using permutational multivariate analysis of variance (PERMANOVA) with 99,999 permutations, implemented using the adonis2 function in vegan.

We also tested for differences in ITS2 sequence diversity among habitats using PCoA and PERMANOVA for *C. pacificum* (based on *psbA* ^ncr^ sequences and ITS2 type profiles dominated by C1d and C1ag) when hosted by *P. tuahiniensis* colonies, and for *C. latusorum* (based on *psbA* ^ncr^ sequences and ITS2‐type profiles dominated by C42a and C42g) when hosted by *P. meandrina* colonies. Only habitats with 10 or more samples were included, which also meant that there were not enough samples for this analysis in other *Pocillopora* species.

To estimate niche breadth, we used the measure proposed by Levins (1968), which estimates the uniformity of the distribution of individuals among resource states (Carscadden et al., 2020). We estimated reef habitat niche breadth by considering resource states to be the five reef habitats, and symbiont niche breadth as the 475 ITS2 sequence types recovered in the post_med output from SymPortal. Niche breadth was then estimated as

$$B=\frac{1}{\sum p_{j}^{2}}$$

And standardized to a scale from 0 to 1 by

$$B_{A}=\frac{\left(B-1\right)}{\left(R-1\right)}$$

where $p_{j}$ is the proportion of individual colonies from a given *Pocillopora* species found in habitat *j*, or the proportion of ITS2 sequence types *j* in each individual colony for a given *Pocillopora* species. A niche breadth of 0 occurs when a species is only found in one habitat, or an individual colony is only associated with one ITS2 sequence type. A niche breadth of 1 occurs when the relative abundance of a species is equal across all habitats, or individual colonies host an equal amount of all ITS2 sequence types.

To estimate niche overlap among cryptic *Pocillopora* species, we used the Horn–Morisita index (Horn, 1966)

$$C_{H}=\frac{2\sum_{i}^{R}p_{\mathrm{ij}}p_{\mathrm{ik}}}{\sum_{i}^{R}p_{\mathrm{ij}}^{2}+\sum_{i}^{R}p_{\mathrm{ik}}^{2}}$$

where $p_{\mathrm{ij}}$ is the proportion of reef habitat, or ITS2 sequence type, *i* used by species *j*. $p_{\mathrm{ik}}$ is the proportion of reef habitat, or ITS2 sequence type, *i* used by species *k*. *R* is the total number of reef habitats or ITS2 sequence types. The Horn–Morisita index is interpreted as the probability that two individuals drawn randomly from each of two species will both belong to the same species, relative to the probability of randomly drawing two individuals of the same species from each species alone (Horn, 1966). The index varies from 0, when the two species are associated with completely different reef habitats or ITS2 sequence types, to 1 when two species are equally associated with exactly the same reef habitats or ITS2 sequence types. Note that the Horn–Morisita measure of overlap measures the extent to which the niche space of species *k* overlaps that of species *j*, so is not symmetrical between species. We chose to present niche breadth and overlap estimates as boxplots to show variation among sites and individuals.

## 3 RESULTS

### 3.1 Relative abundance of cryptic host species (*Pocillopora*) across habitats

**FIGURE 2:**
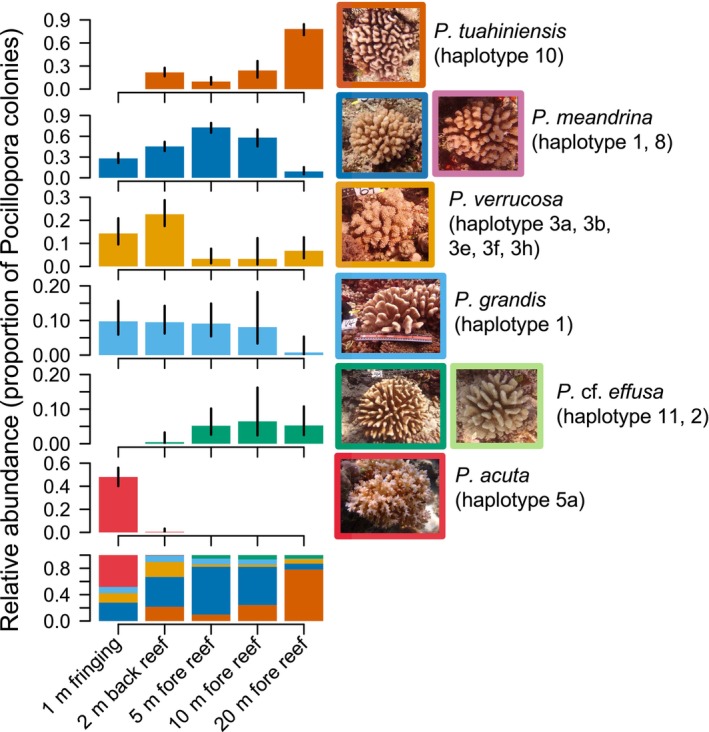
The proportion of all *Pocillopora* spp. colonies belonging to each genetically identified species (rows) in each habitat (*x*‐axis) over four sites around the island of Moorea. Images show examples of gross colony morphologies in the field (but do not necessarily show the morphological variation within species). Haplotypes refer to mtORF haplotypes. Sample sizes are shown in Table 1 and statistical results are shown in Table 3. Note the different *y*‐axis scales between each species. The proportion and 95% confidence interval were estimated from a binomial generalized linear model.

From extensive sampling of 724 *Pocillopora* colonies from around the island of Moorea, there were six sympatric species of *Pocillopora*: *P. meandrina* (*n* = 303, 41.9%), *P. tuahiniensis* (*n* = 182, 25.1%), *P. verrucosa* (*n* = 88, 12.2%), *P. acuta* (*n* = 75, 10.4%), *P. grandis* (*n* = 56, 7.73%), and *P*. cf. *effusa* (*n* = 20, 2.76%). *Pocillopora meandrina*, *P. verrucosa*, and *P. grandis* occurred in all habitats. *Pocillopora tuahiniensis* and *P*. cf. *effusa* occurred in all habitats except the fringing reef. *Pocillopora acuta* exclusively occurred in the fringing reef habitat, except in the back reef lagoon at site 1 where one colony (out of 75) was sampled. The relative abundance differed among habitats in all species (Figure 2; Table 3). The habitat that *P. tuahiniensis*, *P. meandrina*, and *P. verrucosa* were most common differed among each of these species. The patterns of relative abundance across habitats when all sites were pooled generally reflected the patterns within each site (Figure S1). The notable exception was the fringing reef habitat at site 4, in which *P. meandrina*, *P. verrucosa*, and *P. grandis* were relatively more common than *P. acuta*, which dominated the fringing reef habitat of the other three sites.

**TABLE 3:** Statistical results for the effect of habitat on the proportion of *Pocillopora* colonies belonging to each genetically identified species in each habitat from binomial generalized linear models.

Species	Habitats	χ^2^	df	*p*‐Value
*P. tuahiniensis*	Back reef, fore reef 5, 10, 20 m	176.251	3	<0.001
*P. meandrina*	Fringing reef, back reef, fore reef 5, 10, 20 m	152.166	4	<0.001
*P. verrucosa*	Fringing reef, back reef, fore reef 5, 10, 20 m	45.467	4	<0.001
*P. grandis*	Fringing reef, back reef, fore reef 5, 10, 20 m	16.699	4	0.002
*P*. cf. *effusa*	Back reef, fore reef 5, 10, 20 m	13.087	3	0.004
*P. acuta*	Fringing reef, back reef	–	–	–

*Note*: Statistical tests for *Pocillopora acuta* are not included because all but one colony was sampled from the fringing reef.

*Pocillopora tuahiniensis* was relatively more common at 20 m on the fore reef, and its abundance relative to other *Pocillopora* species decreased sharply from only 20 to 10 m. *Pocillopora verrucosa*, to which *P. tuahiniensis* is most closely related phylogenetically and which are morphologically similar, exhibited the opposite pattern, being relatively more common in the back reef lagoon and fringing habitat, while relatively rare on the fore reef. *Pocillopora meandrina*, which cannot reliably be differentiated from P. *tuahiniensis* or *P. verrucosa* in the field, had a “hump‐shaped” relative abundance pattern extending from the shore, peaking at the 5 m fore reef habitat, unlike the other species. *Pocillopora grandis* exhibited no change in relative abundance across habitats, except at 20 m depth on the fore reef where its relative abundance was lowest. *Pocillopora* cf. *effusa*, which most closely resembles *P. grandis* morphologically, was relatively more common on the fore reef at all depths compared to the back reef habitat, and was not present in samples from the fringing reef.

**FIGURE 3:**
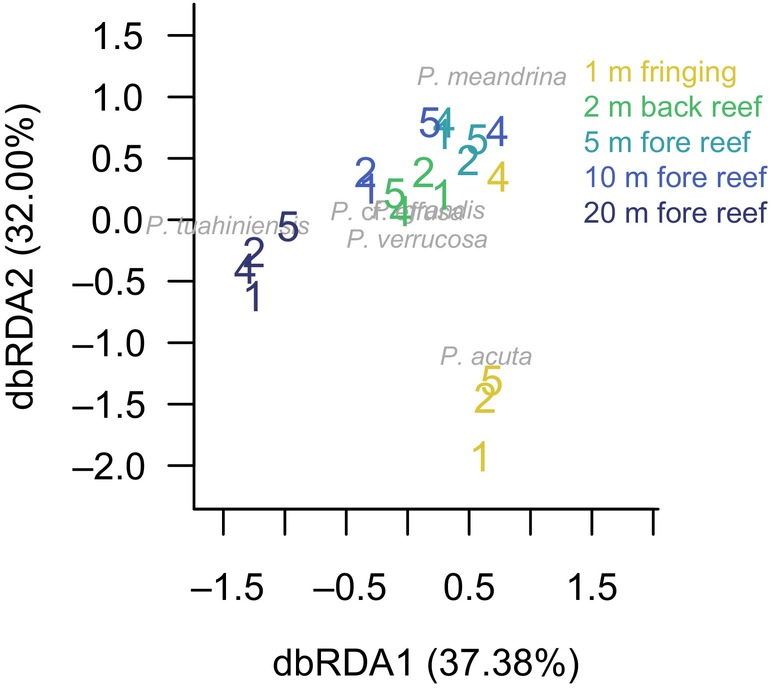
Composition patterns of *Pocillopora* species in each habitat (colors) and site (numbers). Ordination biplot shows the first two axes of a distance‐based redundancy analysis (dbRDA) using the habitat type as the constraining variable. Species scores are plotted in grey, indicating the relative contribution of each species in causing differences in the community composition among the 20 sampling locations.

The difference in relative abundance patterns among species led to the composition of *Pocillopora* spp. varying among habitats (*F*_4,15_ = 10.56, *p* < 0.0001; Figure 3). The *Pocillopora* community was most unique at either end of the habitat gradient: the fringing reef and 20 m on the fore reef (Figure 3). The *Pocillopora* community in the fringing reef was characterized by relatively high abundance of *P. acuta*, the exception being the fringing reef at site 4 (see also Figure S1). The *Pocillopora* community at 20 m was characterized by relatively high abundance of *P. tuahiniensis*. The composition of the *Pocillopora* community was relatively similar across the back reef lagoon, and at 5 m and 10 m on the fore reef, because species whose relative abundance differed the most between back reef lagoon and shallow fore reef habitats (i.e., *P. verrucosa, P*. cf. *effusa*) were relatively rare overall.

### 3.2 Associations between host (*Pocillopor*a) and symbiont (Symbiodiniaceae) identity

**FIGURE 4:**
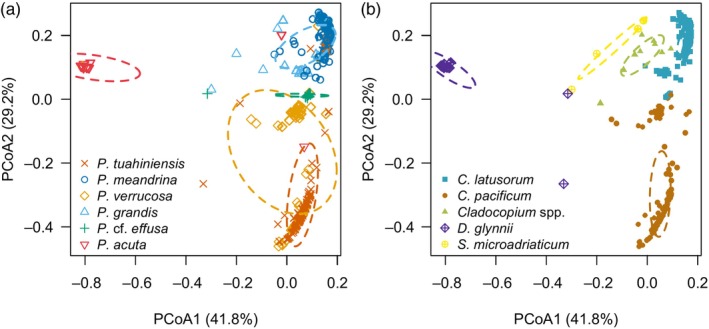
Composition patterns of Symbiodiniaceae based on ITS2 sequence diversity (i.e., the full post‐quality‐filtering diversity of ITS2 sequence variation using the post_med output from SymPortal). Both plots show the same ordination biplot using the first two axes of a principal coordinates analysis (PCoA). Each dot represents a single *Pocillopora* colony. Each colony is identified by its host species identification in (a), and by its dominant symbiont species in (b) based on a combination of *psbA* ^ncr^ sequences and ITS2‐type profiles. Ellipses denote the standard deviation of data around the centroid.

**FIGURE 5:**
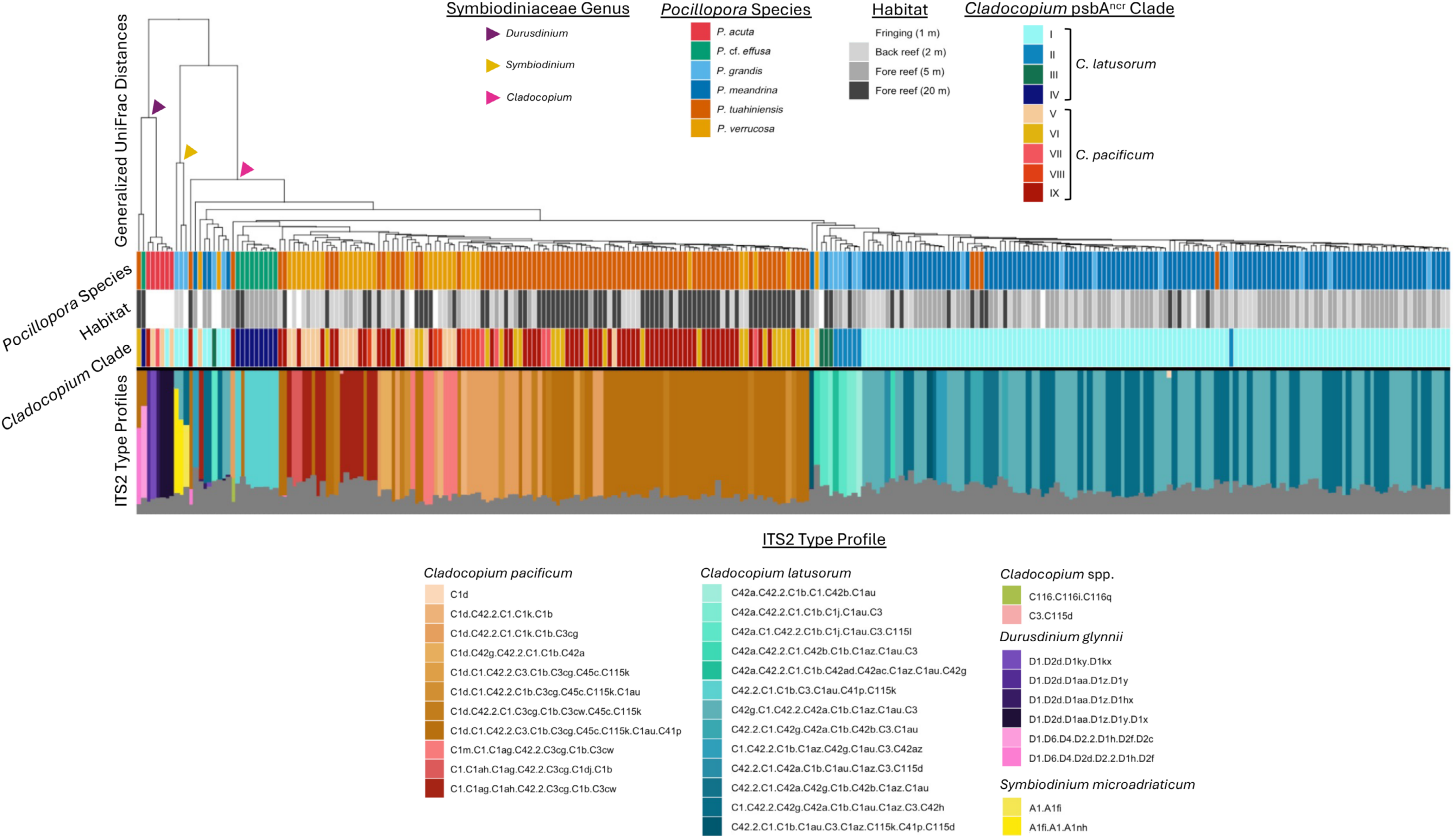
Analysis of samples with both ITS2 sequences (SymPortal post‐med ITS2 sequence data) and *psbA* ^ncr^ sequences. The top tree shows the UPGMA tree of the between‐sample Generalized UniFrac distances from SymPortal. Each vertical bar represents a single coral sample, showing the *Pocillopora* identity derived from the mtORF and RFLP molecular assays (first row), the habitat from which the sample was collected (second row), the hosted clade of *Cladocopium* spp. identified from the *psbA* ^ncr^ sequences (Figures S2 and S3) (third row), and ITS2‐type profiles from SymPortal, colored according to type profile with grey showing the proportion of non‐profile sequences found in each sample (fourth row).

The composition of Symbiodiniaceae, in terms of ITS2 sequence diversity (i.e., the full post‐quality‐filtering diversity of ITS2 sequence variation based on the post_med output from SymPortal) differed among *Pocillopora* host species (*F*_5,371_ = 134.28, *p* = 0.0001; Figures 4 and 5). Using *psbA* ^ncr^, nine *Cladocopium* clades were recovered, four within *C. latusorum* and five within *C. pacificum* (Figures S2 and S3). Clades I through VI were well supported and recovered the clades reported in Johnston, Cunning, et al. (2022) and Johnston, Wyatt, et al. (2022), though clades VII, VIII, and IX were relatively less well supported (all within *C. pacificum*; see Figure S3). *psbA* ^ncr^ *Cladocopium* clades tended to host multiple ITS2‐type profiles, with varying degrees of overlap (Figure S4). ITS2 sequence C1d is usually diagnostic of *C. pacificum* (Turnham et al., 2021), but our data show that ITS2 sequence C1ag can also be considered diagnostic of *C. pacificum* based on comparisons to samples that also had *psbA* ^ncr^ sequences (Figure 5; Figure S4). Specifically, and all in *P. verrucosa*, the six colonies with ITS2‐type profile C1m.C1.C1ag.C42.2.C3cg.C1b.C3cw also had *psbA* ^ncr^ *C. pacificum* clades V or IX, two colonies with ITS2‐type profile C1.C1ah.C1ag.C42.2.C3cg.C1dj.C1b also had *psbA* ^ncr^ *C. pacificum* clades V and IX, and 13 colonies with ITS2‐type profile C1.C1ag.C1ah.C42.2.C3cg.C1b.C3cw also had *psbA* ^ncr^ *C. pacificum* clades V, VIII, and IX (Figure S4).

Based on a combination of *psbA* ^ncr^ sequences and ITS2‐type profiles, 96.79% of *P. meandrina* colonies hosted *C. latusorum*, while the remaining 3.21% hosted *Cladocopium* C116 spp. (which were all from the 5 m fore reef habitat) (Figure 5; Table 4). Samples with C116 ITS2‐type profiles produced poor quality *psbA* ^ncr^ sequences and could not be aligned with those from *C. latusorum* or *C. pacificum*. *Pocillopora* colonies with *Cladocopium* C116 spp. were also found in *P. grandis* (*n* = 1 from 5 m fore reef), *P. tuahiniensis* (one from 5 m and one from 20 m fore reef), and *P. acuta* (*n* = 1, which was the only *P. acuta* colony sampled from the back reef).

**TABLE 4:** Summary of the associations between host *Pocillopora* species and symbiont Symbiodiniaceae species.

	*Cladocopium latusorum* (*psbA* ^ncr^ +ITS2 C42a, C42g)	*Cladocopium pacificum* (*psbA* ^ncr^ + ITS2 C1d, C1ag)	*Cladocopium* spp. (ITS2 C116)	*Symbiodinium microadriaticum* (ITS2 A1, A1fi)	*Durusdinium glynnii* (ITS2 D1, D2d, D6, D4)
*P. meandrina*	96.79% (*n* = 151)		3.21% (*n* = 5)		
*P. grandis*	92.59% (*n* = 25)		3.7% (*n* = 1)	14.81% (*n* = 4)	
*P*. cf. *effusa*	92.31% (*n* = 12)	15.38% (*n* = 2)			7.69% (*n* = 1)
*P. tuahiniensis*	5.71% (*n* = 8)	92.86% (*n* = 130)	1.43% (*n* = 2)		0.71% (*n* = 1)
*P. verrucosa*	4.26% (*n* = 2)	91.49% (*n* = 43)			6.38% (*n* = 3)
*P. acuta*	2.5% (*n* = 1)	15% (*n* = 6)	2.5% (*n* = 1)		95% (*n* = 38)

*Note*: Numbers represent the percent (number) of colonies from each *Pocillopora* species (rows) that hosted a given Symbiodiniaceae species (columns). The identification of Symbiodiniaceae species was based on both *psbA* ^ncr^ and ITS2‐type profiles for *Cladocopium latusorum* and *C. pacificum*, or ITS2‐type profiles for *Cladocopium C116* spp., *Symbiodinium microadriaticum*, and *Durusdinium glynnii*. Percentages for each host species (each row) do not sum to 100% because some colonies hosted two symbiont species (see text for more detail).

92.59% of *P. grandis* colonies hosted *C. latusorum*, three of which also hosted *Symbiodinium microadriaticum* based on ITS2‐type profiles dominated by A1 and A1fi. *Pocillopora* cf. *effusa* hosted *C. latusorum* (92.31% of colonies) or *C. pacificum* (15.38% of colonies) (Table 4). One *P*. cf. *effusa* colony hosting *C. latusorum* (based on *psbA* ^ncr^) also hosted *Durusdinium glynnii*, and one also hosted *C. pacificum* (based on ITS2‐type profiles). The identification of *D. glynnii* was based on both ITS2‐type profiles and on *psbA* ^ncr^ sequences (T. LaJeunesse, personal communication, and Wham et al., 2017).

The majority of *P. tuahiniensis* (92.86%) and *P. verrucosa* (91.49%) colonies hosted *C. pacificum* (Figure 5; Table 4). However, 5.71% (*n* = 8) of *P. tuahiniensis* colonies hosted *C. latusorum*, indicated by both *psbA* ^ncr^ and ITS2‐type profiles in four colonies (Figure 5), and by ITS2‐type profiles in the other four colonies. One *P. tuahiniensis* colony hosting *C. pacificum* (based on *psbA* ^ncr^) also hosted *D. glynnii* (based on ITS2‐type profile). Similarly, 4.26% (*n* = 2) of *P. verrucosa* colonies hosted *C. latusorum*, indicated by both *psbA* ^ncr^ and ITS2‐type profiles in one colony, and ITS2‐type profiles in the other. Three *P. verrucosa* colonies hosted *D. glynnii*, but did not produce *psbA* ^ncr^ sequences.

Based on ITS2 sequences, the majority of *P. acuta* colonies (95%) hosted *D. glynnii* (Table 4), five of which also hosted *C. pacificum* and one also hosted *C. latusorum* (based on *psbA* ^ncr^).

### 3.3 Genetic variation in symbiont species within each host species across habitats

**FIGURE 6:**
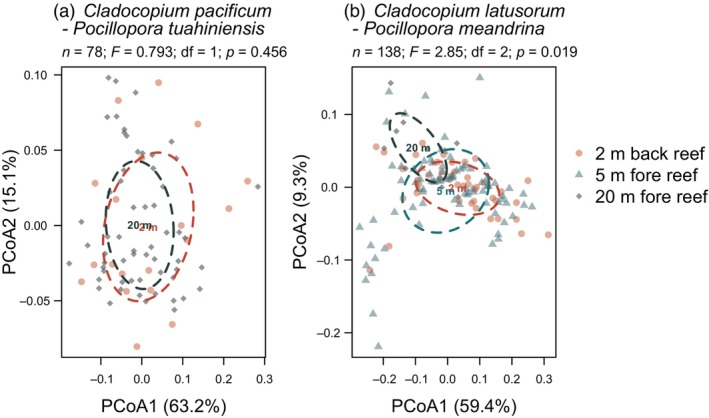
Principal coordinate analyses (PCoA) of Symbiodiniaceae ITS2 sequence diversity among habitats (denoted in legend) for (a) *Cladocopium pacificum* (mostly *psbA* ^ncr^ clades IX and VI) hosted by *Pocillopora tuahiniensis* (*n* = 21 at 2 m, *n* = 59 at 20 m), and (b) *C. latusorum* (mostly *psbA* ^ncr^ clade I) hosted by *P. meandrina* (*n* = 44 at 2 m, *n* = 85 at 5 m, *n* = 11 at 20 m). Sample size (*n*) and PERMAMOVA results (*F* = *F*‐value, df = degrees of freedom, *p* = *p*‐value) are presented at the top of each plot. Ellipses denote the standard deviation of data around the centroid. Note that two outlier samples were removed from each analysis to ensure they were not driving the differences among habitats.

For *C. pacificum* hosted in *P. tuahiniensis*, ITS2 sequence diversity overlapped among colonies sampled from the back reef versus 20 m on the fore reef (Figure 6a). However, ITS2 sequence diversity for *C. latusorum* differed among *P. meandrina* colonies sampled from the back reef and 5 m on the fore reef versus 20 m on the fore reef (Figure 6b).

*Durusdinium glynnii* was hosted by *P. acuta*, and to a lesser extent *P. verrucosa* (Table 4) that were sampled from the fringing reef. From the 20 m fore reef habitat, *D. glynnii* was only found in two colonies (*P. tuahiniensis* and *P*. cf. *effusa*). *Cladocopium* C116 spp. was only found in colonies from the back reef, 5 m fore reef, and 20 m fore reef. *Symbiodinium microadriaticum* was only found in *P. grandis* from shallow habitats: the fringing reef, back reef, and 5 m fore reef.

### 3.4 Niche breadth and overlap

**FIGURE 7:**
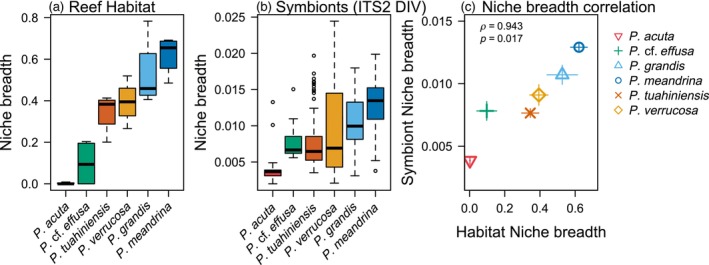
Levin's niche breadth estimates for each *Pocillopora* species in terms of (a) reef habitats and (b) symbionts (ITS2 sequence diversity, DIV). Boxplots show the spread of niche breadth estimates across each of the four sampling sites in (a), and among each individual coral colony in (b) (sample sizes in Table 1). (c) shows the correlation between the species mean reef habitat niche breadth and the species mean symbiont niche breadth. Error bars denote standard error. *ρ* = Spearman's rank correlation coefficient, *p* = *p*‐value.

**FIGURE 8:**
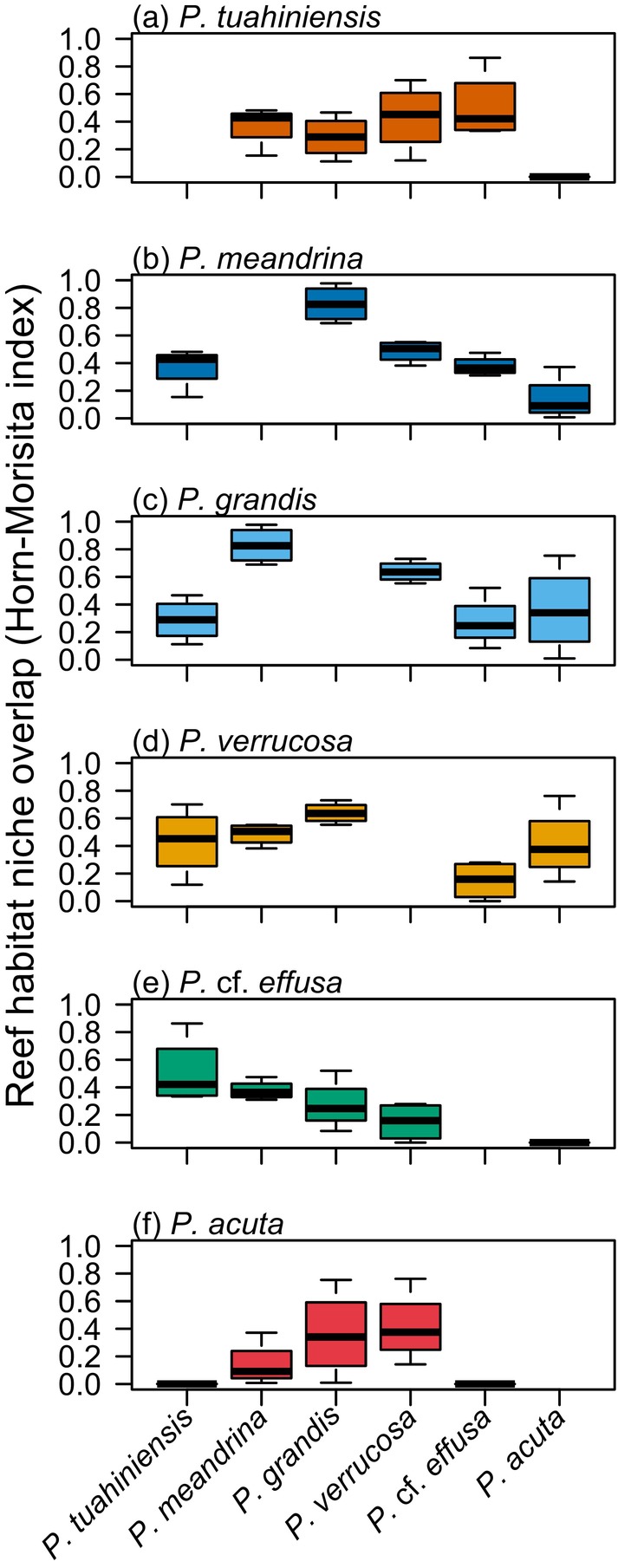
Niche overlap between a given species (a–f) with other species, in terms of its reef habitat niche distribution. Niche overlap is measured using The Horn–Morisita index. Each plot shows the extent to which the relative abundance distribution of that species overlaps with the other species.

*Pocillopora meandrina*, which cannot reliably be differentiated from P. *tuahiniensis* or *P. verrucosa* in the field, had the broadest reef habitat niche breadth (Figure 7), because it was relatively more common in all habitats (Figure 2). *Pocillopora meandrina* had a high niche overlap with its sister species *P. grandis* (Figure 8). *Pocillopora verrucosa* had a similar niche breadth to sister species *P. tuahiniensis*, but the former was most common in shallow habitats (1 m fringing, 2 m back reef) and the latter most common in deep habitats (20 m on the fore reef) (Figure 8). *Pocillopora acuta* had the narrowest niche breadth because it was largely restricted to the fringing reef (Figure 2).

All *Pocillopora* species generally had narrow niche breadths in terms of the symbiont identities they associate with (based on ITS2 sequence diversity). However, the rank order of symbiont niche breadths among *Pocillopora* species was similar to that for habitat niche breadths. As a result, there was a positive correlation between reef habitat niche breadth and symbiont niche breadth across *Pocillopora* species. That is, *Pocillopora* species associating with a broader range of ITS2 DIV's also occupied a broader range of reef habitats.

## 4 DISCUSSION

We present an extensive dataset on the fine‐scale niche differences among morphologically cryptic species, the results of which have important implications for the conservation and restoration of coral populations. By using genetic markers to properly identify species of both the coral host (724 colonies) and the symbiont (from 423 colonies [58%]), we uncovered niche differences among the common group of *Pocillopora* corals that cannot be reliably identified to species in the field based on morphology and have typically been lumped together or misidentified in previous studies. We found that the relative abundance of *Pocillopora* species differed across habitats within the reef—that is, while most species can be found in most habitats, no two *Pocillopora* species had the same distribution–abundance pattern across the same habitats. In general, cryptic *Pocillopora* species have shifted their realized niche space relative to each other while maintaining similar dispersion of niche positions among their individuals. Sister taxa *P. verrucosa* and *P. tuahiniensis* (Johnston & Burgess, 2023; Johnston, Cunning, et al., 2022) had similar niche breadths, and hosted the same specialist symbiont species (mostly *Cladocopium pacificum*), but the former was most common in shallow habitats and the latter most common in deep habitats. In contrast, sister taxa *P. meandrina* and *P. grandis* had overlapping niches and high niche breadths, and hosted the same specialist symbiont species (mostly *C. latusorum*), though *P. meandrina* was much more common overall than *P. grandis*. *Pocillopora* cf. *effusa* tended to host the same symbiont species as *P. meandrina* and *P. grandis*, but exhibited a narrower niche breadth, being relatively more common on the fore reef than the back reef (and absent on fringing reefs). *Pocillopora acuta* is the only brooding species (the other species are broadcast spawners; Schmidt‐Roach et al., 2012) and had the narrowest niche breadth, being restricted to the fringing reef habitat, where unlike other host species, most colonies hosted the generalist, and thermally tolerant, symbiont species *Durusdinium glynnii*. Other species with which *P. acuta* overlapped the most were relatively rare (*P. grandis* and *P. verrucosa*). We also uncovered a relationship between reef habitat niche breadth and symbiont niche breadth across *Pocillopora* species, such that *Pocillopora* species associating with a broader diversity of symbionts (ITS2 sequence diversity) also occupied a broader diversity of reef habitats. Overall, our results support the hypothesis that structural habitat zones within coral reefs, not just depth, play an important role in the generation and coexistence of cryptic *Pocillopora* species and their associated Symbiodiniaceae.

The patterns of relative abundance we uncovered could be driven by several mechanisms that are not mutually exclusive (Grosberg & Levitan, 1992; Hughes et al., 2000). One hypothesis is that larvae are capable of dispersing among all habitats, and all species recruit at equal densities in all habitats, but post‐settlement survival favors different species in different habitats where species traits, including symbiont identity and physiology, best match the local environmental conditions (Edmunds et al., 2024; Prada & Hellberg, 2014; Rippe et al., 2021), such as light and temperature variability (Johnston, Wyatt, et al., 2022). Our results suggest that symbiont identity might not be that important for explaining the relative abundance of hosts (except perhaps in *P. acuta*), since the same symbiont species occurred in hosts with different relative abundance patterns. However, the genetic diversity in symbionts may be an important driver or outcome of the relative abundance patterns of *Pocillopora* hosts. Furthermore, the local environment does not just relate to the physical environment, such that species may have the same fundamental niche, but competition, corallivory, or pathogens in each habitat causes differences in their realized niche after larval dispersal (Howe‐Kerr et al., 2023; Huertas et al., 2021). A second hypothesis is that post‐settlement survival does not differ among species or habitats, but rather a species is relatively more abundant where it has relatively higher larval settlement (Gaines & Bertness, 1992). Species differences in larval settlement among habitats could arise through larval transport, which could occur if species spawn at different times with different current speed and direction, or through larval behavior and habitat selection (Edelaar et al., 2008; Mulla et al., 2021; Schmidt‐Roach et al., 2012). Our preliminary data on recruitment onto settlement tiles suggest that at least *P. acuta*, with brooded larvae and larval dispersal over the scales of meters (Gorospe & Karl, 2013), does not recruit at fore reef locations (Burgess unpublished data). A third hypothesis is that the recruitment and environmental suitability among habitats do not differ among species, but the current distribution reflects the history of disturbance and recovery cycles. We consider this third hypothesis least likely for most species because coral cover was reduced to <1% by 2010 at Moorea and increased steadily from sexual recruitment until 2019 (Edmunds, 2018). However, the marine heatwave in 2019 caused much higher mortality on the fore reef and in *P*. cf. *effusa* compared to that in other *Pocillopora* species (Burgess et al., 2021). Prior to 2019, it was likely that *P*. cf. *effusa* was at least equally dominant with *P. meandrina* and *P. tuahiniensis* on the fore reef (Burgess et al., 2021). Therefore, an important question now is understanding the relative contribution of multiple processes facilitating niche partitioning (e.g., Johnston, Wyatt, et al., 2022).

Although *C. latusorum* and *C. pacificum* are mostly host specialists, similar to that reported previously (Johnston, Cunning, et al., 2022; Turnham et al., 2021), our results show that, by sampling hundreds of colonies and using multiple genetic markers, there is more flexibility than has previously been documented. Sister species *P. tuahiniensis* and *P. verrucosa* tended to host *C. pacificum*, but some colonies instead hosted *C. latusorum* (verified by both *psbA* ^ncr^ and ITS2 sequences), while other colonies also hosted *D. glynnii*. Both *Pocillopora* species are thought to transmit symbionts vertically (from parent to offspring) (Schmidt‐Roach et al., 2012), but the presence of *C. latusorum* and the generalist species *D. glynnii*, suggest that at least some horizontal acquisition (from the environment) is possible (Quigley et al., 2017). Similarly, *Pocillopora* spp. colonies hosting *C. latusorum* also hosted *S. microadriaticum* and *D. glynnii*, and colonies hosting *C. pacificum* also hosted *D. glynnii*. Overall, there was evidence that habitats structured genetic variation (based on ITS2 sequences) of *C. latusorum* algae hosted by *P. meandrina*, but not for *C. pacificum* algae hosted by *P. tuahiniensis*. A similar finding was reported in the Great Barrier Reef at larger scales (across latitudes), where *C. latusorum* hosted by *P. meandrina* exhibited a “north” and a “south” lineage that corresponded to the bifurcation of the South Equatorial Current at 16°S, whereas *C. pacificum* hosted by *P. verrucosa* (sister taxa to *P. tuahiniensis*) did not exhibit multiple lineages across ~2000 km (Marzonie et al., 2024).

Our results highlight the importance of sampling across the relevant spatial scales and environments (Connolly et al., 2005; Karlson & Cornell, 1998). Most previous studies have focused on factors structuring cryptic diversity and symbiosis by sampling across large spatial scales, such as across the entire ocean basins (Glynn et al., 2023; Oury et al., 2023; Voolstra et al., 2023) or across several degrees of latitude (Buitrago‐López et al., 2023; Marzonie et al., 2024; Matias et al., 2023; Meziere et al., 2024). The comparison of samples across islands and reefs over such large scales could be confounded if a species is not sampled because it was rare in the particular habitat that was sampled, or if different habitats are sampled among reefs. Although sampling a standardized habitat at each reef can avoid within‐reef patterns confounding among‐reef patterns, it will still miss species that are rare in that particular habitat but present at that reef or island, giving misleading estimates of geographic range and diversity. Substantial environmental variation occurs within reefs (Guadayol et al., 2014; Johnston, Wyatt, et al., 2022; Reid et al., 2019; Wyatt et al., 2020, 2023), and ignoring such fine‐scale environmental variation among habitats or depths within reefs is likely to provide an incomplete understanding of how the environment affects the distribution of genetic and species diversity, and symbioses, that are relevant to understanding acclimatization and adaptation. Furthermore, sampling *Pocillopora* species based on in situ morphological assessment will provide an incomplete picture at the species level, not only because such sampling will likely reveal multiple genetic species within a morphological group (Oury et al., 2023, Voolstra et al., 2023), but it will miss individuals that are the same genetic species but not sampled because they were morphologically different. What might be considered a large, widespread species based on morphological indicators, may instead be many species with populations that are smaller, geographically restricted, specialized to different habitats within reefs, and more prone to extinction than previously thought (Meziere et al., 2024).

The presence of cryptic species specialized to different habitats within a reef system has particularly important implications for coral restoration. The most common method of coral restoration involves transplanting fragments of coral colonies to the reef, either directly from existing fragments or from coral gardening (where small fragments are raised in nurseries prior to outplanting) (Boström‐Einarsson et al., 2020). Corals in the genus *Pocillopora* are one of the most common corals used in restoration (Boström‐Einarsson et al., 2020; Hughes et al., 2023), but are often identified as species based on morphology, or are all considered as a single species. Especially because most restoration programs target a particular morphological group (Hughes et al., 2023), unknowingly collecting or growing fragments from a species with the highest capacity for survival and reproduction in fore reef habitats and attempting to use those fragments to restore populations in back reef habitats, for example, will be less successful than using a species most suited to back reef habitats. Similarly, using fragments from a cryptic species that is particularly susceptible to bleaching (e.g., *P*. cf. *effusa*; Burgess et al., 2021) would lead to decreased success rates, especially if heatwaves were responsible for depleting coral populations in the first place. Restoration practices that translocate corals or their offspring among habitats and ignore cryptic species are also prone to unintentionally reconfiguring species–environment specializations and manipulating gene flow that could negatively affect near‐future adaptive capacity, and ultimately do more harm than good. On the other hand, restoration programs that actively target cryptic species and deliberately incorporate diverse habitats are likely to have higher success because a given morphological group will contain the available portfolio of genotypes, rather than a narrow subset, which will be especially important when the particular species or genetic variants that will survive and reproduce in the near future is unpredictable (Colton et al., 2022; Webster et al., 2017). It is, therefore, critical that restoration practitioners use sound knowledge and principles to maximize positive outcomes, even if restoration is at best only likely to be successful at very small spatial and temporal scales (Hughes et al., 2023).

Our results also have important implications for understanding how temporary micro‐refuges within reefs could underlie recovery at reef scales. Disturbances that threaten coral reefs, such as heatwaves, cyclones, Crown‐of‐Thorns seastar outbreaks, pollution, and over‐fishing occur over regional scales but are also spatially variable at small scales within reefs (Baird et al., 2018; Crosbie et al., 2019; Penin et al., 2007; Sully & van Woesik, 2020; Yadav et al., 2023). Especially in relation to coral bleaching, small‐scale environmental “refuges” associated with depth (Baird et al., 2018; Crosbie et al., 2019; Penin et al., 2007), distance from shore (Matias et al., 2023; Sully & van Woesik, 2020), or back reef pools (Bay & Palumbi, 2014; Thomas et al., 2018) are common. For example, the marine heat wave at Moorea (French Polynesia) in 2016 affected corals on the back reef much more than the fore reef (Donovan et al., 2020), whereas the heat wave in 2019 affected corals on the fore reef much more than the back reef (Burgess et al., 2021). Such differences are likely mediated by higher nutrients in the back reef compared to the fore reef (Leichter et al., 2013), and the dynamics of depth‐dependent internal wave cooling on the fore reef (Wyatt et al., 2020, 2023). The implications of our results are that habitat‐specific disturbances or environmental changes will affect some cryptic species more than others, which could lead to resilience via response diversity (Baskett et al., 2014; Burgess et al., 2021), or simply elevate the extinction risk in vulnerable species in certain habitats. Such habitat–abundance associations would also lead to species responses to habitat‐specific disturbances that are potentially independent of any intrinsic differences in the physiological responses among different species. Furthermore, it will be difficult to discern whether differences in the response to the environment or disturbance among habitats at the genus level (i.e., without genetically identifying colonies to species and lumping them together) are driven by environmental conditions of the habitat or the cryptic species that dominates there.

Our ability to anticipate the consequences of climate change and restoration interventions will depend on understanding how genetic diversity is partitioned among species that are morphologically indistinguishable but ecologically and evolutionarily distinct (Colton et al., 2022; McManus et al., 2021; Webster et al., 2017). In that light, the identification and habitat associations of cryptic species and their algal symbionts here is important because we have uncovered niche differences within one of the most common group of corals (*Pocillopora*) that cannot be reliably identified to species in the field based on morphology and have typically been lumped together or misidentified in the past. An important implication is that researchers using transplant experiments between depths or habitats could misinterpret their results as being caused by depth or habitat, when they are instead due to differences among cryptic species. Similarly, assessment of treatment effects on a purported species of *Pocillopora* will be misleading if analyses are not based on the identification of colonies to species using genetics. Previous results on the ecology of *Pocillopora* at the species level should be considered unreliable if samples were not genetically identified to species, and future studies should endeavor to genetically identify samples to species when studying the population and community ecology of *Pocillopora*.

## FUNDING INFORMATION

This work was funded by a National Science Foundation grant to S.C. Burgess (OCE 1829867).

## CONFLICT OF INTEREST STATEMENT

The authors declare no conflict of interest.

## Supporting information


Appendix S1

## Data Availability

All data, sequences for mtORF, ITS2, and *psbA* ^*ncr*^, and R code used to reproduce the analyses and plots can be found at Dryad https://doi.org/10.5061/dryad.wh70rxwx4 or Zenodo https://zenodo.org/doi/10.5281/zenodo.12745665.
