# Supplementary material for: Niche breadth and divergence in sympatric cryptic coral species (Pocillopora spp.) across habitats within reefs and among algal symbionts
Source: Evol Appl. 2024 Aug 2;17(8):e13762. doi: 10.1111/eva.13762 (PMC11294925; doi:10.1111/eva.13762)
Supplement: Supplementary file 1 — Appendix S1 [file EVA-17-e13762-s001.docx]

**Supplemental Figures**

**Suppl. Fig. 1:** The proportion of all *Pocillopora* spp. colonies belonging to each genetically identified species (rows) in each habitat (x-axis) and site (columns). Images show examples of gross colony morphologies in the field. Haplotypes refers to mtORF haplotypes. Samples sizes are shown in Table 1 and statistical results are shown in Table 3 of the main text. Note the different y-axis scales between each species (rows). Sites Pooled shows the proportion and 95% confidence interval estimated from a binomial generalized linear model.

**Suppl. Fig. 2:** Schematic of the Bayesian phylogenetic tree based on psbA^ncr^ sequences for *Cladocopium* spp, showing the identity and position of clades within *C. latusorum* and *C. pacificum*. Clade identity matches those shown in Fig. 5 of the main text.

**Suppl. Fig. 3:** Bayesian phylogenetic tree of *Cladocopium* spp. psbA^ncr^ sequences showing the identity and position of clades within *C. latusorum* and *C. pacificum*. Black and white circles at nodes indicate where support was >95% CI. Clade identity matches those shown in Fig. 5 of the main text and Supplemental Fig. 2. psbA^ncr^ Clade matching from Johnston et al. (2022) to current paper is as follows: I = I; II = II; III = IV, IV = V; V = VI.

**Suppl. Fig. 4:** Relationship between *psbA^ncr^ Cladocopium* clades and ITS2 type profiles. n indicates that number of samples. The vertical black line denotes *C. latusorum* clades to the left (I – IV) and *C. pacificum* clades to the right (V – IX).
